# Case of Cerebro Spinal Meningitis

**Published:** 1877-05-20

**Authors:** E. W. Lane

**Affiliations:** Georgia


### SOUTHERN MEDICAL RECORD.

Vol. VII. ATLANTA, GA., MAY 20, 1877. No. 5.

THOMAS S. POWELL, M.D.,

W. T. GOLDSMTIH, M. D.,

R. C. WORD, M. D.,

} Editors.

R. C. WORD, Business Manager.

SUBSCRIPTION: TWO DOLLARS PER ANNUM, IN ADVANCE.

☞ All communications, and letters on business connected with The Record for 1877 must be addressed to the Business Manager.

### Original and Selected Articles.

### CASE OF CEREBRO SPINAL MENINGITIS.

By E. W. LANE, M. D., of Georgia.

Thinking it might interest some of your numerous readers, I send you report of a case of *cerebro spinal meningitis*, which occurred the latter part of August, 1876. My little son, aged 15 years, had, for two or three days, complained of soreness in his legs and arms, and was puffed about his eyes. The night previous to his attack, he was troubled with nightmare, and slept but little. At breakfast he ate but little, and complained of slight headache, but went to school as usual. About 3 o'clock P. M., my wife received a message that he was very sick, and went, with horse and buggy, to bring him home. She found him suffering with severe headache and high fever, with occasional spasmodic contractions of the upper extremities. Shortly after she got him home, I came in, and found him convulsed—Opisthotonos well marked. I was not long in making out my diagnosis, and proceeded at once to combat the formidable enemy. As soon as his muscles were sufficiently relaxed, and he could swallow, I gave him xv grs. of calomel with j gr. of podophylin, shaved both temples and scarified them well, and applied cups until I drew about six ounces of blood. I then applied cold water to his head by pouring it from a pitcher held at one or two feet distant from his head, and suffering it to fall in a small stream, until several gallons had been poured on his head, when he expressed himself as feeling better. Soon after I stopped the water, his head began to get hot, and in a few minutes he had another convulsion. After it passed off, which was several minutes, I applied a mustard plaster about two inches wide over the whole length of the spinal column, and gave him 20 drops of Tincture Gelseminum

Sempervirens. In about half an hour afterwards, he had another convulsion, after which I gave him 30 drops more. In half an hour, he again had a slight convulsion. I repeated the dose by giving 40 drops more, and in about an hour his muscular system became perfectly relaxed, he perspired freely, and fell asleep. He slept about two hours, when he awoke with another convulsion, though not so bad as at first. I gave him 30 drops more, and continued to give it in 20 drop doses every two hours for about five days, during which time, whenever, by neglect, or forgetfulness, we would suffer him to come from under the influence of Gelseminum, the convulsive symptoms would return, but a single dose would allay them. I gave no other medicine. Gave an injection of soapsuds once daily to procure an action on his bowels, and occasionally would pour cold water on his head when it became very hot—frequently applied cold, wet cloths to his forehead. He was delirious four days and nights, talking foolishly; had to be watched to keep him on his bed. On the sixth day he began to show signs of returning reason, and on the seventh could answer questions and ask for water. He continued to mend slowly, and at the end of three weeks was convalescent, though very weak. His hair all fell out, but, by the aid of tonics, has recovered his health, grows finely, and his mind perfectly good.

[The above case is interesting as proving the great anti-spasmodic powers of gelseminum, and the safety with which under some circumstances large doses oft repeated may be borne. It is a fine febrifuge, and quinia will be well borne during the febrile exascubation if each dose be combined with ten or fifteen drops of the gelseminum.—Eds.]

